# The feasibility of a behavioral group intervention after weight-loss surgery: A randomized pilot trial

**DOI:** 10.1371/journal.pone.0223885

**Published:** 2019-10-21

**Authors:** Michelle R. Lent, Laura K. Campbell, Mackenzie C. Kelly, Jessica L. Lawson, Jessica M. Murakami, Sasha Gorrell, G. Craig Wood, Marianne M. Yohn, Stephanie Ranck, Anthony T. Petrick, Krystal Cunningham, Megan E. LaMotte, Christopher D. Still

**Affiliations:** 1 Clinical Psychology, Philadelphia College of Osteopathic Medicine, Philadelphia, Pennsylvania, United States of America; 2 Geisinger Clinic, Geisinger, Danville, Pennsylvania, United States of America; Weill Cornell Medical College in Qatar, QATAR

## Abstract

**Background:**

Formal psychosocial support programs after weight-loss surgery are limited in scope and availability.

**Objective:**

This randomized pilot study evaluated the feasibility of a postoperative behavioral intervention program.

**Materials and methods:**

Postoperative weight-loss surgery patients (*N* = 50) were recruited from February 2017–July 2017 and randomized to a four-month behavioral program or usual care wait-list. Outcomes evaluated in addition to feasibility included health-related quality of life (Short Form -36), psychosocial functioning and adherence. Secondary outcomes included within-group changes for each outcome.

**Results:**

Out of eight possible sessions, intervention participants attended a mean of 4.2 sessions. Intervention group participants experienced greater improvements in the social functioning domain of health-related quality of life compared to usual care. Self-reported dietary adherence in the intervention group remained stable, while usual care group dietary adherence declined. Within the intervention group, participants also reported gains in the physical function, pain and general health aspects of quality life from baseline to post-treatment. No differences in weight, mood or other eating behaviors (e.g., loss of control, emotional eating) were evident between groups.

**Conclusion:**

Though participation in a postoperative behavioral intervention varied, the program helped participants to maintain aspects of quality of life and self-reported adherence to dietary recommendations.

**Trial registration:**

ClinicalTrials.gov NCT03092479

## Introduction

Weight-loss surgery is an effective treatment for severe obesity [1], but long-term success requires adherence to a regimented postoperative diet and lifestyle [2]. Numerous behavioral and psychosocial factors can make adherence to the recommended diet and lifestyle challenging [3–6], but little psychological or behavioral treatment beyond support groups is available as standard of care after surgery [2]. Psychiatric medication use is higher in bariatric surgery patients than in the general population [7] and two-thirds of patients have a lifetime diagnosis of at least one psychiatric disorder [8]. Modifiable psychosocial and behavioral factors, including depression [3], binge eating [9], low levels of social support [4] and high life stress [4] may contribute to difficulties with postoperative adherence, and in turn, to the occurrence of weight regain [10, 11]. Further, patients frequently cite low quality of life [12] secondary to obesity as a reason to pursue bariatric surgery; however, untoward psychosocial changes such as substance use problems [13], marital discord [14] and fluctuations in mood [14] can manifest after surgery and diminish expected gains in quality of life. The causes of these changes remain unclear, but may include variations in the metabolism of substances postoperatively [15, 16] or changes in interpersonal dynamics that can occur after weight loss.

Previous approaches to postoperative behavioral or psychosocial programs, though limited in number, include acceptance-based [17, 18], cognitive-behavioral and/or dialectic-behavioral [19, 20], structured dietary interventions [21] and brief strategic therapies [22]. A review of postoperative psychological interventions reported a positive relationship between these programs and weight loss after bariatric surgery [23]. Similarly, a meta-analysis of randomized, controlled trials (RCTs) of behavioral programs in bariatric surgery also found behavioral treatment to relate to greater weight loss, but highlighted the need for evaluation of outcomes beyond weight loss, such as psychosocial functioning after surgery [24]. Notably, health-related quality of life was not a focus of the five lifestyle intervention RCTs included in this meta-analysis [24], which varied greatly in duration (3–36 months) and sample size (30–144 participants).

To date, the majority of studies of postoperative behavioral intervention programs focus on the prevention of weight regain [18, 24] and very few are randomized, controlled trials. The objective of this randomized, controlled pilot trial was to evaluate the feasibility of a postoperative behavioral group intervention program in weight-loss surgery patients. Outcomes measured also included psychosocial functioning and adherence. This intervention targeted patients that completed surgery within 18 months to provide treatment and skill acquisition before significant weight regain typically begins [5, 10, 25]. We hypothesized that psychosocial functioning, health-related quality of life, and adherence (diet and physical activity) would be significantly higher in patients randomized to the behavioral intervention program compared to usual care.

## Materials and methods

This study was approved by the Geisinger Institutional Review Board. All participants provided informed consent.

Adult (≥ 18 years) weight-loss surgery patients from a large, integrated rural health system were recruited to participate in this prospective, randomized controlled trial from February 2017 to July 2017. The preoperative program for weight-loss surgery candidates is a six to twelve-month multidisciplinary clinical curriculum consisting of medical and psychosocial evaluation, as well as diet, nutritional and lifestyle education with a goal of 10% presurgical body weight loss to promote a reduction in liver fat and greater surgical safety. As part of standard clinical care, patients attend appointments with a psychologist, bariatrician, surgeon, nurse and dietitian, among others. Following the psychosocial evaluation, patients are provided a “light status” indicating clearance status for weight loss surgery from the program’s Behavioral Medicine team (i.e., “green light” = cleared for surgery; “yellow light” = delayed clearance and behavioral goals). Reasons for delayed clearance (“yellow light”) typically include difficulty making dietary and other behavioral changes, poor understanding of the procedure, risks or associated lifestyle changes, or untreated disordered eating or mental health concerns. Postoperatively, patients are scheduled for follow-up visits at one week, two weeks, two months, five months, eight months, and twelve months after surgery, and then yearly thereafter.

Patients were eligible to participate if they were a minimum of 18 years old, completed primary weight-loss surgery (Roux-en-Y gastric bypass [RYGB], sleeve gastrectomy [SG], or biliopancreatic diversion [BPD]/duodenal switch [DS]) within the past 18 months. The 0–18 month postoperative period was selected for the intervention to impact change in quality of life and adherence before most patients reach their weight-loss nadir. Patients who underwent surgery revisions, were pregnant, or cognitively unable to provide informed consent were excluded. Eligible patients were identified using electronic health records and mailed a recruitment letter in random order. Letters were followed by up to three scripted phone calls by research staff. Participants who expressed interest were then invited for a screening visit and to provide written informed consent.

Patients considering bariatric surgery at the study site undergo preoperative psychological evaluations to evaluate their readiness for surgery. All patients are then given a “traffic light” status (red, yellow, or green) according to their readiness. Patients given a “green” light are cleared for surgery, while “yellow” light patients have preoperative milestones that require further attention before surgery. It is plausible that yellow light patients may experience greater benefit from the intervention. Therefore, participants were stratified by initial psychosocial evaluation status (cleared initially (“green light” or cleared after achieving behavioral goals “yellow light”) and then randomized to intervention or usual care (1:1) using a random number generated computer program provided by the study statistician. Recruitment, randomization and treatment occurred in two waves. Recruitment efforts included all eligible patients, regardless of initial light status or whether they previously expressed interested in postoperative behavioral treatment. The authors confirm that all ongoing and related trials for this intervention are registered. Internal review board approval of the trial listing, as well as requested edits by the registry, resulted in a one-month gap between enrollment initiation and registry listing. The protocol is available at dx.doi.org/10.17504/protocols.io.2bagaie.

### Intervention

The behavioral intervention program consisted of eight, one-hour group sessions that occurred twice monthly and included up to 10 postoperative bariatric surgery patients (8 sessions over 16 weeks). The intervention schedule was selected to allow for adequate time to deliver content while minimizing patient burden of travel to the medical center. Three waves of groups were co-led by a licensed clinical psychologist or doctoral-level psychology trainee. Trainees were provided regular supervision by the attending psychologist to review the previous session, offer ongoing training in leading groups, and to provide guidance regarding delivery of subsequent session content. Intervention content, timing and duration were evidence-based [5, 21, 24] and based on cognitive-behavioral therapy (CBT) principles. Content focused on coping with potential psychosocial changes after surgery and developing skills for postoperative diet and adherence. Examples of specific topics discussed in the group intervention include problem-solving potential barriers and challenges managing food cravings, mindful eating, triggers for emotional eating, managing special occasions and changes to routine. Patients collaboratively set goals for diet, physical activity, adherence and other behavioral changes tailored to the needs of each participant. Sessions began with a review of participant progress on their goals and homework from the previous session, as well as problem solving barriers encountered (approximately 30 minutes). The remainder of each session focused on introducing and discussing new material. Participants were asked to develop a new action plan for the next two weeks and to state their goals at the end of each session. Details of the intervention are in Table 1. Intervention group participants were reimbursed for travel expenses ($15) and time ($50) to complete study visits (total $205 possible). Given the pilot nature of this study, we did not require participants to attend a minimum number of sessions to be included in analyses.

**Table 1:** Cognitive-behavioral intervention session content and materials.

Session	Topic(s)	Patient Materials
1	**Introduction and Preventing Malnutrition** - Introduction, format and group norms - Motivation for weight-loss surgery and program participation - Postoperative nutrition, fluid and vitamin recommendations - Non-adherence risks: malnutrition, dehydration, hair loss, etc. - Predicting and problem-solving adherence barriers	1. Blank Weight Chart 2. Weekly Food & Activity Logs 3. Group Norms 4. Treatment Outline 5. Home Practice–Self-monitoring 6. Program Contacts 7. Timing of Meals and Snacks
2	**Addressing Problematic Eating Patterns (Part 1)** - Review of homework - Grazing and loss of control over eating - Cognitive-behavioral model - Behavioral strategies for eating (self-monitoring, stimulus control, contingency management, meal planning) - Eating-related thoughts, behaviors and emotions, including cognitive distortions and cravings	1. BASICS of Mindful Eating 2. Home Practice: - Self-Monitoring - Mindful Eating
3	**Addressing Problematic Eating (Part 2)** - Review of homework - Self-care - Psychoeducation about emotions - Emotional eating cycle	1. Emotional Eating–Coping with Stress 2. Home Practice: - Self-Monitoring - Self-care
4	**Physical Activity for Health, Wellness and Weight Loss Maintenance** - Review of homework - Psychoeducation on benefits of physical activity - Setting physical activity goals - Planned versus daily routine activity	1. Physical Activity Worksheet 2. Home Practice: - Physical Activity Goal
5	**Managing Special Occasions** - Review of homework - Motivation for weight loss and value-driven behavior - High-risk situations: Identification, planning and strategies - Self-weighing	1. Strategies for Planning Ahead 2. Values 3. Home Practice: - Planning Ahead
6	**Relationships and Social Support After Surgery** - Review of homework - Social adjustments postoperatively - Partner adjustments postoperatively - Enhancing support and assertive communication	1. Assertive Communication 2. Home Practice
7	**Body Image** - Review of homework - Psychoeducation on body image - Self-compassion strategies	1. Self-Appreciation and Compassion 2. Home Practice: - Write a letter to your body
8	**Taking Stock and Maintaining Progress** - Identifying lapses: “slips” and “falls” - Relapse prevention strategies/skills - Recognizing mental health concerns - Review and recap	1. Cognitive Distortions Worksheet 2. Problem Solving Nutrition Challenges 3. Taking Stock and Maintaining Progress

### Usual care

Usual care patients completed questionnaires only. Upon study completion, usual care patients were offered the behavioral treatment provided to the intervention group after all study data were collected. Usual care participants were reimbursed for time ($50) to complete study visits ($100 total possible).

### Measures

Self-report questionnaires were collected at baseline following consent and again four-months later. Intervention patients completed questionnaires following their final group or were mailed surveys if unable to attend the final session. Control patients were offered the option of completing follow-up surveys during a study visit or via mail.

#### Short form-36 (SF-36)

The SF-36 [26–28], developed for the RAND Medical Outcomes Study (MOS), is a valid and reliable measure previously used in populations with obesity. The SF-36 was used in this study to measure health-related quality of life (HRQL). The SF-36 has eight quality of life scales (physical functioning, role limitations [physical, emotional], energy, emotional well-being, social functioning, pain and general health), as well as a total score (range 0–100).

#### Patient health questionnaire-9 (PHQ-9)

The PHQ-9 [29] is a brief screening tool for depression severity. Scores of five = mild depression, ten = moderate, and fifteen or more = severe (range 0–27).

#### State-trait anxiety inventory (STAI)

The STAI [30] is a forty-item assessment of anxiety symptoms and is scored on a four-point Likert scale (; range 20–80 per subtest).

#### Weight efficacy life-style (WEL, Short Form)

The WEL [31] is an eight-item measure that assesses self-efficacy in resisting the urge to eat in various situations (range 0–80).

#### Loss of control over eating scale-brief (LOCES)

This seven-item measure evaluates loss of control over eating over the past twenty-eight days and is scored on a five-point Likert scale (; range 5–35) [32, 33].

#### Emotional eating scale (EES)

The EES [34] is a twenty-five-item scale that evaluates the urge to eat while experiencing a range of emotions in three domains: anger, anxiety and depression (subscale score range 0–36 or 0–44).

#### Dietary adherence

Dietary adherence was measured by nine-point Likert scale (“How well are you following the diet plan given to you by the dietitian?”). This question was used in a previous weight-loss surgery study (range 1–9) [35].

#### Physical activity adherence

The Paffenbarger Physical Activity Questionnaire (PPAQ) [36]is an eight-item questionnaire designed to measure participation in physical activity by asking about the number of city blocks walked, stairs climbed and the duration and frequency of planned sporting and recreational activities. We calculated minutes per week of Moderate to Vigorous Physical Activity (MVPA) by adding block per day (assume 3.0 MPH speed) and physical activity of at least moderate level [37].

#### Weight change

Weight change was calculated as percentage total body weight change from time of enrollment to end of treatment or waist-list control period (%WL). Weight was obtained using the weight closest to the enrollment and study conclusion available in the medical record.

#### Process outcomes

Mean number of group attendees and attendance were tracked to determine treatment tolerability and feasibility.

To reduce the chance of data entry errors, the questionnaire responses were manually entered into a database and then visually verified against the source documents by a researcher who did not enter the original data. SAS software (Version 9.4, Cary, NC) was used to evaluate outliers and electronically score questionnaires with standard scoring algorithms.

### Analyses

The demographic and baseline characteristics of the randomized groups were described using percentages and means with standard deviations and were compared between groups using Fisher’s exact tests and two-sample t-tests. A similar analysis was conducted to determine if there were differences between participants that completed study procedures (completers) versus those that did not complete study procedures (non-completers). Change in questionnaire scores was evaluated descriptively within each group. Cohen’s *d* (defined as the mean difference between groups divided by the pooled standard deviation; small effect = 0.2, medium = 0.5, large = 0.8) was used to provide a common effect size for change in scores from questionnaires of various scales. To explore whether the initial psychosocial evaluation light status modified the effect of the intervention, change in questionnaire scores was compared between intervention participants with an initial “green light” versus intervention participants with an initial “yellow light.”

## Results

### Participants

**Fig 1:**
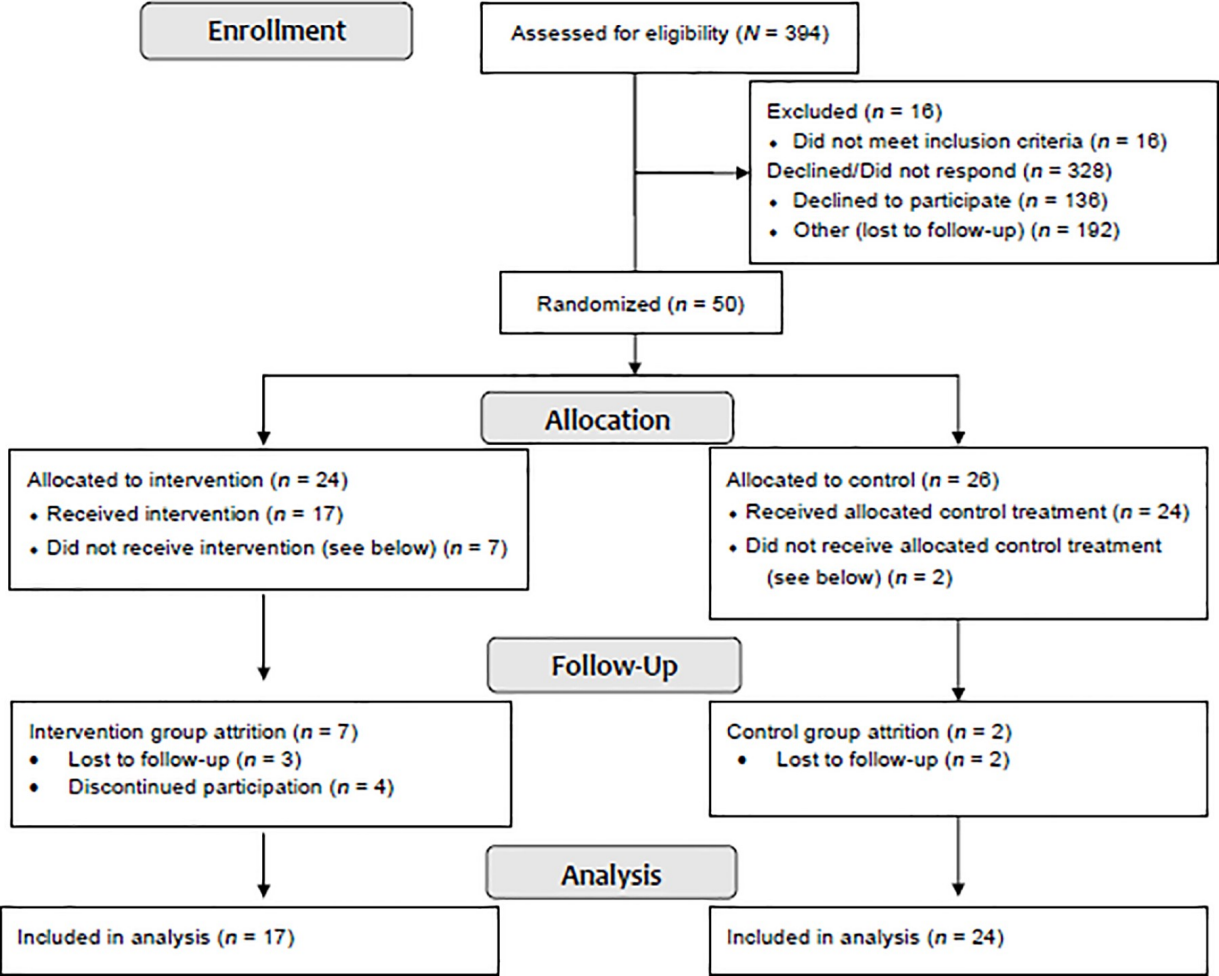
Participant flow CONSORT diagram.

Of the 378 eligible weight-loss surgery patients, 50 patients enrolled and were randomized (13.2%) to the intervention (*n* = 24) or usual care (*n* = 26). The majority of participants were female (82%), White (94%) and underwent RYGB (66%, Table 1). The mean time from surgery to enrollment was 7.0 months (*SD* = 3.8). Approximately half of participants (*n* = 28) received psychosocial clearance in one visit (“green light”, Table 1) and the remaining (*n* = 22) were cleared following completion of behavioral goals (“yellow light”). Intervention and usual care group participants did not differ at baseline (Table 2, *p* > 0.05). Patients who declined to participate most frequently cited distance to the Health System and lack of time or conflicting interests as obstacles preventing enrollment (given the rural setting of the Health System, many patients travel long distances for appointments). Participant flow through this clinical trial is in Fig 1.

**Table 2:** Baseline characteristics of weight-loss surgery patients (*N* = 50).

	Intervention (*n* = 24)	Control (*n* = 26)	*p*-value
**Sex** Female, % (*n*)	83% (*n* = 20)	81% (*n* = 21)	0.99^1^
Male, % (*n*)	17% (*n* = 4)	19% (*n* = 5)	
**Race** White, % (*n*)	96% (*n* = 23)	92% (*n* = 24)	
Black, % (*n*)	0% (*n* = 0)	4% (*n* = 1)	0.99^1^
Hispanic, % (*n*)	4% (*n* = 1)	4% (*n* = 1)	
**Age** (years) mean (*SD*) [range]	47.6 (9.1) [27, 58]	46.2 (12.0) [18, 64]	0.67^2^
**Body Mass Index** (BMI) kg/m^2^ mean (*SD*) [range]	47.1 (6.7) [38.9, 64.1]	50.4 (6.2) [41.8, 65.1]	0.07^2^
Surgery type*: RYGB, % (*n*)	71% (*n* = 17)	62% (*n* = 16)	
SG, % (*n*)	29% (*n* = 7)	27% (*n* = 7)	0.35^1^
PD/DS, % (*n*)	0% (*n* = 0)	12% (*n* = 3)	
**Initial Psychosocial Clearance Light status:** Green, % (*n*)	54% (*n* = 13)	54% (*n* = 15)	0.99^1^
Yellow, % (*n*)	46% (*n* = 11)	46% (*n* = 11)	
**Baseline questionnaire scores:**			
SF36-Overall, mean (*SD*)	70.4 (17.9)	63.3 (18.9)	0.18
STAI-S, mean (*SD*)	45.0 (5.5)	44.6 (6.1)	0.80
STAI-T, mean *(SD)* PHQ-9, mean (*SD*)	45.7 (6.4) 5.5 (4.5)	45.2 (6.1) 7.5 (5.8)	0.71 0.19
LOCES, mean (*SD*)	11.1 (4.1)	13.0 (5.3)	0.17
WELSF, mean (*SD*)	59.3 (15.8)	59.0 (17.4)	0.95
EES–Frustration, mean (SD) EES–Anxiety, mean (SD) EES–Depression, mean (SD)	8.2 (8.1) 8.4 (6.6) 5.6 (3.9)	8.5 (8.8) 6.8 (6.8) 5.5 (4.8)	0.88 0.39 0.98
Dietary adherence, mean (*SD*)	7.1 (1.3)	7.4 (1.2)	0.43
PPAQ (MVPA min/week), mean (*SD*)	120.8 (177)	82.3 (135)	0.39
**Months from surgery to enrollment:**			
Mean (*SD*)	7.0 (3.6)	6.9 (4.1)	0.93

^1^Fisher’s exact test

^2^two-sample t-test

*RYGB = Roux-En-Y Gastric Bypass, SG = Laparoscopic Sleeve Gastrectomy, PD/DS = Biliopancreatic Diversion /Duodenal Switch

Of the 50 randomized participants, 82% (*n* = 41) completed study procedures (intervention: n = 14 completed treatment and n = 17 completed questionnaires at month four; control: n = 24 completed questionnaires at month four). When comparing completers versus non-completers, there was no difference in race, age, BMI at the time of surgery, weight-loss surgery procedure type, initial psychosocial evaluation light status, and most of the baseline questionnaire summary scores (*p* > 0.05). However, a higher percentage of females completed study procedures as compared to males (females 87.8%, males 55.6%, *p* = 0.043) and completers had lower overall mean quality of life score (64.2 versus 79.2, *p* = 0.035), lower mean anxiety score (STAI total: 88.9 versus 96.9, *p* = 0.039), and higher physical activity adherence (PPAQ: 110.4 versus 48.8, *p* = 0.028). Although the rate of completion was higher in the usual care group, the difference was not significant (70.8% intervention, 92.3% usual care, *p* = 0.069). The primary reasons for attrition were travel time to the Health System and competing obligations (family, work, etc.).

### Health-related quality of life

At study conclusion, patients in the intervention group experienced greater improvements in the social functioning domain of HRQL (+12.5±18.8 versus -0.5±19.0 points) compared to usual care (Cohen’s *d* = 0.69, Table 3).

**Table 3:** Comparison of change in questionnaire responses between intervention and control participants.

	Change from Pre to Post		Cohen’s *d*	Results favor:
	Intervention (*n* = 17)	Control (*n* = 24)		
**Health related QL**				
SF36-Physical function	13.5 (11.7)	6.3 (15.2)	0.521	Intervention
SF36-Physical limitations	20.6 (45.3)	14.6 (37.5)	0.147	-
SF36-Emotional limitations	11.8 (55.2)	1.4 (25.0)	0.258	-
SF36-Energy	10.0 (20.0)	4.2 (17.5)	0.314	-
SF36-Emotional well being	4.9 (14.2)	1.8 (11.9)	0.247	-
SF36-Social functioning	12.5 (18.8)	-0.5 (19.0)	0.690	Intervention
SF36-Pain	10.0 (18.1)	0.7 (27.3)	0.389	-
SF36-General health	10.3 (15.7)	1.4 (14.0)	0.608	Intervention
SF36-health change	-1.5 (16.5)	3.3 (20.4)	0.251	-
SF36-Overall	11.4 (15.4)	4.6 (12.1)	0.503	Intervention
**Mood**				
STAI-S-anxiety	0.9 (4.9)	0.3 (6.0)	0.106	-
STAI-T-anxiety	-1.5 (4.1)	-0.4 (4.7)	0.248	-
PHQ-9	-0.9 (4.6)	-1.1 (4.5)	0.053	-
**Eating behaviors**				
LOCES	0.1 (3.9)	-0.7 (3.5)	0.224	-
WELSF	2.0 (14.1)	-1.6 (10.9)	0.294	-
EES-Frustration	1.5 (7.1)	0.1 (7.3)	0.183	-
EES-Anxiety	-0.4 (7.0)	1.9 (6.1)	0.352	-
EES-Depression	1.5 (4.8)	0.0 (3.8)	0.347	-
**Dietary Adherence**				
Dietary adherence	0.3 (0.8)	-0.8 (1.4)	0.927	Intervention
**Physical Activity Adherence**				
PPAQ (MVPA min/week)	71 (160)	-14 (145)	0.564	Intervention

Limited to the subset of participants that completed the pre- and the post-questionnaires

Within the intervention group, participants experienced improvements in physical functioning, social functioning, pain, general health and overall HRQL pre- to post-treatment (Table 3). No significant changes in HRQL from study initiation to conclusion within the usual care group were found.

### Mood

No noteworthy differences were found between groups in regards to scale scores on the STAI or PHQ-9 scale scores at study conclusion (Table 3) and changes from pre- to post-treatment were not likely to reflect clinical significance within either group (Table 4). Mean depression scores at baseline and post-treatment across groups were in the mild range.

**Table 4:** Within-group changes* in outcomes in weight-loss surgery patients.

	Intervention (*n* = 17)		Control (*n* = 24)	
	Pre	Post	Pre	Post
**Health related QL**				
SF36-Physical function	74.1 (20.3)	87.6 (14.7)	61.3 (28.4)	67.6 (28.2)
SF36-Physical limitations	63.2 (40.6)	82.8 (36.4)	55.2 (39.0)	69.8 (37.6)
SF36-Emotional limitations	68.6 (46.4)	80.4 (35.5)	72.2 (36.3)	73.6 (39.3)
SF36-Energy	51.2 (27.2)	61.2 (19.2)	44.2 (19.0)	48.3 (24.4)
SF36-Emotional well being	69.9 (18.0)	74.8 (16.6)	70.8 (21.1)	72.5 (20.5)
SF36-Social functioning	70.6 (27.9)	83.1 (20.2)	74.0 (28.3)	73.4 (30.7)
SF36-Pain	59.1 (22.5)	69.1 (20.6)	62.1 (26.5)	62.7 (32.1)
SF36-General health	58.8 (21.5)	69.1 915.0)	60.4 (16.7)	61.8 (19.6)
SF36-health change	92.6 (11.7)	91.2 (19.6)	82.6 (24.3)	85.9 (19.7)
SF36-Overall	66.8 (18.6)	78.1 (16.3)	62.3 (19.0)	66.9 (21.9)
**Mood**				
STAI-S-anxiety	43.8 (5.8)	44.8 (5.8)	44.1 (6.2)	44.5 (5.4)
STAI-T-anxiety	44.7 (4.8)	43.2 (5.9)	45.0 (4.6)	44.6 (5.4)
PHQ-9	6.0 (5.1)	4.9 (4.5)	7.7 (6.0)	6.8 (4.9)
**Eating behaviors**				
LOCES	11.0 (4.5)	11.1 (3.9)	13.1 (5.3)	12.4 (4.9)
WELSF	60.1 (16.2)	62.1 (13.4)	57.8 (17.5)	56.2 (19.8)
EES-Frustration	8.5 (8.0)	9.9 (7.1)	9.5 (8.9)	9.7 (7.6)
EES-Anxiety	9.4 (6.7)	8.9 (5.8)	7.4 (7.0)	9.3 (6.2)
EES-Depression	5.6 (3.6)	7.1 (4.6)	6.0 (4.8)	6.1 (3.9)
**Dietary Adherence**				
Dietary adherence	7.1 (1.5)	7.4 (1.3)	**7.4 (1.3)**	**6.6 (1.6)**
**Physical Activity Adherence**				
PPAQ (MVPA min/week)	147 (200)	218 (239)	84 (140)	70 (132)

*Limited to the subset of participants that completed the pre-questionnaire and the post-questionnaires

### Eating behavior

No differences were found between groups or within group in loss of control over eating (LOCES), urges to eat in various places (WEL) or emotional eating (EES) (Table 3, Table 4).

### Dietary adherence

Intervention patients reported stable adherence to the postoperative diet (+0.3±0.8) pre- to post-treatment, while usual care patients reported declines in adherence from baseline (-0.8±1.4; Cohen’s *d* = 0.927, Table 3).

### Physical activity adherence

Mean change in MVPA did not differ between groups (Table 3).

### Light status

Within the intervention group, the initial psychosocial evaluation light status (initial “green light” versus “yellow light”) was not correlated with change in overall HRQL (*p* = 0.54), changes in dietary adherence (*p* = 0.60) or changes in physical activity (*p* = 0.65).

### Weight change

There was no difference in %WL at the study conclusion between groups (control = -27.2% ± 7.6%, intervention = -30.5% ± 8.1%, p = 0.14).

### Process outcomes

Mean attendance per group was 4.2 participants. Participants had a mean of 1.4 missed groups. One make-up session was offered.

## Discussion

As hypothesized, participation in a behavioral intervention program following weight-loss surgery resulted in higher improvements in aspects of HRQL compared to usual care. Additionally, participation in a behavioral intervention program helped participants to sustain their adherence to postoperative dietary recommendations.

Specifically, weight-loss surgery patients who completed a four-month, group-based intervention program reported better social functioning (e.g., time and extent to which physical health or emotional problems interfere with social activities) compared to usual care controls. Gains in social functioning elevated intervention participants to levels that were comparable to normative populations [28, 38] and reflected a moderate effect size of the intervention. Further, the magnitude of the social functioning improvements from pre to post-treatment (17.7%) were likely indicative of clinically meaningful change [38]. In the intervention group, participants also reported notable increases in overall HRQL, as well as HRQL measures of pain (i.e., amount, impact on activities), general health (i.e., overall health ratings, tendency to getting sick) and physical functioning (i.e., limitations in physical activity and daily living). Our study findings indicate that participation in a postoperative behavioral intervention program has the potential to further enhance the gains in HRQL that typically accompany weight-loss surgery [39].

Dietary recommendations after weight-loss surgery typically include promoting consumption of water, protein and low-calorie foods, while limiting or eliminating intake of high calorie foods and beverages, alcohol and caffeine [2]. Dietary adherence ratings in both groups were comparable and high prior to study initiation. While intervention group dietary adherence remained relatively stable pre- to post-intervention, dietary adherence declined in usual care patients. These findings suggest that a postoperative psychosocial intervention does not enhance adherence, but instead helps weight-loss surgery patients remain “on track” with dietary changes and avoid the deterioration in adherence seen in the wait-list control group.

Physical activity did not differ between groups. Notably, however, intervention participants reported a mean of 218 minutes per week of MVPA at the end of treatment versus only 70 minutes per week of MVPA in usual care participants, which is likely clinical meaningful. While no official recommendation exists regarding amount of physical activity recommended following weight-loss surgery, exercise is an important aspect of the postoperative lifestyle [2]. Notably, this study used self-report measure of physical activity, which can be subject to inaccuracies compared to objective measures [37].

This study’s behavioral program differed from traditional bariatric surgery support groups in modality (cognitive-behavioral psychotherapy versus supportive), target audience (post-surgery patients versus mixed pre- and postoperative patients), frequency (bi-monthly versus monthly) and content (psychosocial functioning + adherence versus adherence alone). A recent review [5] of psychosocial and behavioral interventions (individual or group therapy, support groups) after weight-loss surgery concluded that both psychosocially-based interventions and support groups were associated with enhanced weight reduction. Given the focus on HRQL, and that most participants were still in the acute postoperative weight-loss phase, weight loss was not the primary outcome of this pilot study. No differences between groups were found in weight change or maladaptive eating behaviors, including loss of control, urges and emotional eating, which were topics included in the intervention content. It is possible that the impact of the intervention on weight and eating behaviors would become more evident after patients reached their weight loss nadir and are then at risk for weight regain.

Finally, levels of anxiety or depression between intervention and usual care participants were similar. Additionally, participants who experienced greater challenges obtaining psychosocial clearance prior to surgery (“yellow light”) did not benefit more from the intervention than participants cleared in one session (“green light”). The small sample sizes, however, limited the ability to detect differences by initial clearance status.

This pragmatic clinical trial had several strengths, including the randomized controlled design, the use of an empirically-supported intervention, and the evaluation of outcomes beyond weight loss or regain. Weight-loss surgery can convey a wide range of benefits beyond weight reduction and requires lifelong behavioral changes for lasting success. Psychosocial interventions such as the program evaluated in the present study emphasize an approach to care that encompassed the “whole” patient.

This study has several limitations. First, this was a pilot trial and therefore we were underpowered to detect differences between groups and were limited to presenting effect sizes only. Future studies should include larger samples in more diverse cohorts. Second, our cohort was predominantly White, limiting generalizability of our findings to patients of other races. Timing of enrollment in the study postoperatively varied, which may have influenced the rate of weight loss as well as the degree of psychosocial and quality of life changes. More patients dropped out of the intervention arm than usual care, and intervention participants missed 1–2 sessions on average, which likely relate to the greater burden of time and travel required for participation. Subsequent studies could more comprehensively assess the reasons for missed sessions or attrition; specifically, whether participant burden or dissatisfaction with intervention content related to reductions in engagement. Additionally, most study outcomes were self-reported, which can be subject to bias. The self-report measures selected were chosen to best capture the intent of the intervention, the wide variety in postoperative clinical recommendations of weight-loss surgery programs across the country, and the iterative nature of postoperative clinical guidelines. Subsequent studies could utilize food diaries or standardized 24-hour recalls to obtain greater specificity on dietary adherence. Participants were not blinded to condition and gains may relate to attention given the intervention group had more frequent interaction with the Health System. Finally, study findings were limited by feasibility-related challenges, including reaching expected participation targets, as well as the differential attrition rates in the intervention versus usual care group and the brief intervention period (four months). Reasons cited for treatment refusal or early withdrawal were distance to the Health Center and competing time commitments. Future studies could assess the efficacy and feasibility of minimizing patient burden by delivering behavioral interventions via telephone or online, as was done by ProjectHELP [18] and Kalarchian and colleagues or by offering the program in affiliated satellite locations in patients’ communities in addition to main surgical centers [21, 40]. Future studies may also benefit from longer treatment durations that provide support after patients reach their weight-loss nadir and are at risk for weight regain.

In conclusion, this behavioral intervention resulted in promising potential psychosocial and behavioral benefits for weight-loss surgery patients, particularly in the domains of postoperative lifestyle adherence and HRQL. However, the feasibility of administering postoperative behavioral programs remains unclear. While most weight-loss surgery patients experience improvements in mood and quality of life postoperatively [41], the potential for psychosocial outcomes of concern is becoming increasingly apparent [13, 42]. Implementing structured behavioral intervention programs as standard of care could further elevate gains in functioning that are inherently present for most patients after surgery, as well as provide support and surveillance for patients at risk for adverse outcomes.

## Supporting information

S1 File CONSORT checklist. (PDF)

S2 File IRB approved protocol. (PDF)
