# Supplementary material for: The feasibility of a behavioral group intervention after weight-loss surgery: A randomized pilot trial
Source: PLoS One. 2019 Oct 21;14(10):e0223885. doi: 10.1371/journal.pone.0223885 (PMC6802820; doi:10.1371/journal.pone.0223885)
Supplement: S2 File — (PDF) [file pone.0223885.s002.pdf]

Study Application (Version 1.11)

1.0 General Information

***Please enter the full title of your study:**

A Randomized Trial of a Pilot Behavioral Support Intervention After Bariatric Surgery

***Please enter a short descriptor that you would like to use to reference the study:**

Behavior Study
* This descriptor allows you and other study team members to quickly identify the study. This would be the acronym/ sponsor protocol and/or abbreviated study title.

Please identify the Research Type?

Nutrition/Weight Mgement

2.0 Add Department(s)

2.1 List departments associated with this study:

Primary Dept?	Department Name
○	**GMC** - Adult Psychiatry
◉	**GMC** - Gastroenterology & Nutrition

3.0 Assign key study personnel(KSP) access to the study

3.1 *Please add a Principal Investigator for the study:

Campbell, Laura K, PhD

3.2 If applicable, please select the Research Staff personnel:

A) Additional Investigators

Lent, Michelle R, PhD
Co-Investigator/ Sub-Investigator

B) Research Support Staff

Cunningham, Krystal
Research Assistant
Gorrell, Sasha C
Participating Clinician
LaMotte, Megan E
Research Assistant

Seiler, Jamie L, PA-C
Project Manager
Tittel, Laura
Participating Clinician
Wood, G. Craig, M.S.
Biostatistician
Yohn, Marianne M
Project Coordinator

3.3 *Please add a Study Contact:

Cunningham, Krystal
LaMotte, Megan E
Lent, Michelle R, PhD
Yohn, Marianne M

The Study Contact(s) will receive all important system notifications along with the Principal Investigator. (e.g. The project contact(s) are typically either the Study Coordinator or the Principal Investigator themselves).

3.4 If applicable, please select the Designated Department Approval(s):

Add the name of the individual authorized to approve and sign off on this protocol from your Department (e.g. the Department Chair or Dean).

3.5 If applicable, please select the Administrative Assistant(s):

Administrative Assistant Note

4.0 Basic Review Questions

4.1 Are you reporting an emergency use of an investigational drug or device?

○ Yes ◉ No

4.2 Does the study involve research that may be deemed Exempt? (Prior to checking, please be sure to review the exemption categories outlined in the Help Link)

○ Yes ◉ No

4.3 Does the study <u>ONLY</u> involve use of <u>existing</u> medical records, databases, specimens, etc. (i.e. already on the shelf or collected on or before submission of the study to the IRB)?

○ Yes ◉ No

<u>Please note</u>: Only check YES if the proposed research <u>only</u> involves use of <u>existing</u> (already on the shelf or collected at the time of IRB submission) patient-related information from medical records (both electronic and paper), databases and/or discarded specimens.

<u>Please note:</u> Do not check YES if the study involves <u>prospective</u> data or specimen collection.

4.4 Does the proposed research examine nursing processes, practices, or nursing theory? (ie, studies regarding interventions, measures, outcomes)

( ) Yes (•) No

NOTE: Nurse-initiated research (nurse serving as PI or Co--I) is reviewed and tracking through the Nursing Research Council (NRC).

Designated NRC Unique Tracking Number:

☐ Research proposals are acknowledged by NRC when conducted by a nurse with earned doctorate
☐ Research proposals require NRC approval when conducted by a nurse without an earned doctorate.

5.0 Initial Review Application: Health and Biological Sciences

5.1 Geisinger believes research is important and should be visible to the community and patients. After approval of your study, information about your research study will be included in "Find a Study" on Geisinger's public website. "Find a Study" includes specific contact information for direction of questions or interest in the study. Please enter the following information that should be included for the study:

Enter name of study contact for "Find a Study":

Marianne Yohn

Enter study contact email for "Find a Study":

mmyohn2@geisinger.edu

Enter study contact phone number for "Find a Study":

570-271-6516

Describe the study's target population:

Geisinger patients that completed bariatric surgery within the past 18 months will be eligible to participate.

Select the gender of the target population:

( ) Male
( ) Female
(•) Both

Describe the target age range of the study:

18-65

Please add any other information or additional comments that you would like to include about this study on "Find a Study":

5.2 Location of the Research

☑ Geisinger facility
☐ Non-Geisinger facility

Name of facility:

Geisinger Medical Center

Please attach a letter of support from the non-Geisinger facility.

5.3 Please summarize the proposed study using lay (non-technical) language that can be easily understood by the general public.

<u>PLEASE NOTE:</u> The summary will also be used to describe your study in "Find a Study" on geisinger. org.

Bariatric surgery patients may experience significant psychosocial changes after surgery, but little psychological support is available beyond support groups postoperatively. We will evaluate the effect of a postoperative support program targeting quality of life, psychosocial functioning and adherence to behavior change in Geisinger Health System bariatric surgery patients.
This prospective, randomized pilot trial will evaluate a comprehensive postoperative behavioral support intervention using a 4-month bi-weekly program in 40 bariatric surgery patients from the GHS Center for Nutrition and Weight Management, compared to 40 usual care patients that completed bariatric surgery within one year. The primary outcome will be the difference in quality of life (as measured by the Short Form-36) between groups, as well as differences in psychosocial functioning (mood, eating behaviors) and adherence (diet, physical activity, appointments). Secondary outcomes will include patient satisfaction, treatment feasibility and attrition. Outcomes will be assessed at baseline and treatment completion (4 months). The intervention will focus on addressing psychosocial changes after surgery, strategies for postoperative diet and adherence, and preventing weight regain. Patients will collaboratively set goals for diet, physical activity, adherence and other behavioral changes tailored to the needs of each participant. Intervention patients will attend 8, one-hour bi-weekly treatment sessions in a 4-month time period.

5.4 Identify how this research study is funded.

View Details	Sponsor Name	Sponsor Type
⊟	Clinic Research Fund	Geisinger

Sponsor Name:	Clinic Research Fund
Sponsor Type:	Geisinger
Sponsor Role:	Funding
**Grant/Contract Number:**	
**Is Institution the Primary Grant Holder:**	No

PLEASE NOTE: If there is no funding for the study or if the study is departmentally funded, please select "Geisinger Clinic" as the Sponsor. Regardless of funding, there is a cost to conducting all research, e.g. personnel time; therefore, the clinical department (Geisinger) is supporting the research activity by paying the salary of staff who spend time working on it.

If the study has received external or internal (Geisinger Research Fund) grant funding, a copy of the grant application must be uploaded with the submission.

5.5 Indicate the level of risk associated with this study.

- (•) Minimal Risk
- ( ) Greater Than Minimal Risk

Describe all reasonably expected risks, harms, and/or discomforts that may apply to research. Discuss severity and likelihood of occurrence. ***Consider the range of risks, including physical psychological, social, legal, and economic.***

1. Loss of privacy

2. Emotional distress

Describe how risks, harms and/or discomforts will be minimized. If testing will be performed to identify individuals who may be at increased risk (e.g., pregnant women, individuals with HIV/AIDS, depressive disorders, etc.), address timing and method of testing: include how positive tests will be handled.

1. PHI will be stored on a secure, password protected network to which only limited number of research staff will have access. For data analysis, all data will be de-identified to the extent possible. Any information made available to other investigators will be in a HIPAA compliant format.

2. The group leader (a clinical psychology resident) will assess the participant immediately after group and consult with the study PIs (Dr. Campbell or Dr. Lent). The group leader and study PI(s) will refer the participant for further treatment as appropriate. The group leader may also ask disruptive participants to leave group at any time.

If the study involves greater than minimal risk, provisions for safety monitoring are required to protect participants. Please indicate below the plan for monitoring safety in this study:

- ☐ Data Safety Monitoring Board (DSMB)
- ☐ Data Safety Monitoring Committee (DSMC)
- ☐ PI and Study Staff
- ☐ Other

If Other, please define:

5.6 Please identify any benefits that may be reasonably expected to result from the research, either to the subject(s) or to society at large.

We hope that patients randomized to participate in group sessions will experience improved quality of life and be able to adhere to the recommended after surgery diet and lifestyle. There is no direct benefit for patients randomized to the control group; however, knowledge gained from this study has the potential to benefit future bariatric surgery patients.

Payment to subjects is not considered a benefit in the risk benefit assessment.

5.7 Does your study involve any of the following?

- ☐ Drugs, Biologics or Dietary Supplements
- ☐ Device(s)
- ☑ Not applicable

5.8 Human Subjects - a living individual about whom an investigator (whether professional or student) conducting research obtains: (1) Data through intervention or interaction with the individual, or (2) Identifiable private information.

How many subjects do you plan to enroll:

80

How many people do you estimate that will go through the consent process (but not necessarily enroll) to get the planned “enrolled” subjects?

200 to approach about consent our of a recruitment of 400

All records and/or subjects who are reviewed and/or approached for this research study should be included toward the total number of subjects even if they have no further participation in the study (i.e. drop out, screen out, choose not to participate).

Indicate the total number of subjects to be enrolled across all sites:

N/A

Is this a multi-site study?

○ Yes ◉ No

If yes, are you the lead researcher of the multi-site study?

○ Yes ◉ No

If yes, please include the following specific details in the protocol on how the following will be managed or outline specific details in the box below:

- **Unanticipated problems involving risk to participants or others**
- **Interim results**
- **Protocol modifications**

Describe populations to be excluded from the research. ***Please describe procedures to ensure equitable selection of subjects***

Exclusion criteria includes: pregnancy, revision of bariatric sugery, and significant cognitive impairment that prevents informed consent.

Will vulnerable populations be targeted in the research?*(check all that apply):*

- ☐ Minors (< 18 years of age)
- ☐ Decisionally Impaired
- ☐ Students
- ☐ Geisinger employees
- ☐ Pregnant Women/Fetuses)
- ☐ Prisoners
- ☐ Other

If Other, please specify:

If vulnerable populations are included, please provide a description of the additional safeguards that are used to protect the vulnerable population's rights and welfare.

Provide rationale and justification for the inclusion of each special vulnerable population in the research. Please note "vulnerable" populations require special consideration by the federal regulatory agencies and by the IRB.

Age Range

- ☐ 0-6 years
- ☐ 7-17 years
- ☑ 18+ years

5.9

Please upload all proposed recruitment materials, e.g. advertisements, bulletin board notices, telephone scripts, and recruitment letters for all planned types of media (printed, radio, electronic, TV, or Internet). Each recruitment item must be labeled for its particular use and MUST have a footer containing an identifier, version number, and date. Recruitment flyers must be provided in the final format for approval.

5.10 Please check all applicable recruitment forms.

- [ ] Ad (print)
- [ ] Ad (radio-provide script, then tape)
- [ ] Ad (TV-provide script, then video)
- [ ] Brochure
- [ ] E-mail notice
- [ ] Flyer
- [ ] Information sheets (before study)
- [ ] Information sheets (on study)
- [ ] Internet
- [x] Mass Mailing
- [ ] No recruitment materials will be used
- [ ] Physician to Physician Letter
- [ ] Physician referral
- [x] Records (e.g. medical, employment, school)
- [ ] Recruitment Script (to aid in consent process)
- [ ] Registry or Bank
- [ ] Student Subject Pool
- [x] Subject Letter
- [x] Telephone script/guidance
- [ ] Other

If Other, please describe:

5.11 Initial Contact

Explain who will approach subjects to take part in the research and what will be done to protect the subject's privacy in this process:

Recruitment letters will be mailed to potentially eligible patients determined through the data pull done by the data analyst. Those patients will be called by a member of the study team to discuss the study in more detail, answer any questions, and if interested, a study visit will be scheduled to review the consent form in detail. Data pull information will be kept on password protected computers that only the research team has access to. When patients come in for the study visit to go over the consent, patients will be in an exam room with a member from the research team to answer any questions about the study.
If the support group/class or individual appointment approach is used, a member of the research team will present the information in either a closed group session/class or in an exam room. If anyone in the group/class session has questions that they would like to discuss in private accommodations will be made.

Initial contact of subjects identified through records search must be made by the official holder of the record, i.e. primary care physician, therapist, and public school official.

5.12 Screening and Recruitment

Are you requesting waiver of HIPAA authorization for recruitment purposes?

◉ Yes ○ No

*If **yes**, please complete the Partial Waiver of Authorization for Recruitment.*

5.13 Does the study require the use of tests, procedures, clinic space, clinic visits, professional fees, lab services, pharmacy services or hospital services in order to answer the research question(s)?

○ Yes ◉ No

If yes, a copy of the study schema and billing determination prepared by the Office of Sponsored Programs (OSP) and approved by Office of Research Compliance (ORC) must be uploaded with the submission.

5.14 Compensation and costs of participation

Will subjects receive any compensation or inducements before, during, or after participation in the study (i.e. money, gifts, gift certificates)?

◉ Yes ○ No

If, yes, please check all the appropriate types and reason for the reimbursement or inducement.

- [x] Monetary
- [ ] Non-Monetary
- [ ] Time

If monetary, please provide the amount and timing of payments to participants.

All patients (both Intervention and Control groups) will be compensated $50 each time they fill out surveys (which will be at two time points). They will fill out baseline surveys after they sign the informed consent and have baraitric surgery, and then fill out surveys again 4 months later or at session 8. Patients that are randomized into the intervention group will be reimbursed $15 for the other 7 group sessions they attend. These sessions will be held twice a month for 4 months. The total amount that the intercentional group could possible get is $205, and the total amount that the control group could possible get is $100 because they are not attending any sessions until agter the study is over and if they desire to just as a courtesy/benefit to them.

If non-monetary, please describe:

If other, please define:

5.15 How will informed consent/assent be obtained for the study? ***Please check one of the following***

◉ Written Consent
○ Waiver of written documentation of consent (Verbal consent from subjects will be obtained)
○ Waiver of consent (no consent from subject will be obtained.)

5.16 Confidentiality of Data

Explain how information is handled, including storage, security measures (as necessary), and who will have access to the information. Include details for both electronic and "paper" copy records.

An electronic database will be created and data will be entered into the database by research assistants. The database will be password protected and paper surveys kept in a locked file drawer. Each participant

will be assigned a unique study ID number. A list of study ID numbers with associated identifying information (e.g., name, medical record number) will be electronically on a secure, password protected server. Access to the identifiers will be limited to study team members that require use of the identifiers. All paper informed consents and surveys will be collected by the research team and kept in a locked cabinet.

Explain if any personal or sensitive information that could be potentially damaging to participants (e.g., relating to illegal behaviors, alcohol or drug use, sexual attitudes, mental health, etc.) will be collected.

Please provide details regarding retention of research records (including identifiable data) at the end of the study. For instance, how long and where will the research records and data be maintained.

Study records will be retained for 6 years. After 6 years, the study-specific database will be deleted and paper surveys shredded.

Please check all the PHI elements that will be received by the study team:

- [x] Names (first name, last name, or initials)
- [x] Geographic subdivisions smaller than a state
- [x] Dates
- [x] Telephone numbers
- [ ] Fax numbers
- [x] E-mail addresses
- [x] Social security numbers
- [x] Medical record numbers
- [ ] Health plan beneficiary numbers
- [ ] Account numbers
- [ ] Certificate/license numbers
- [ ] Vehicle identifiers and serial numbers, including license plate numbers
- [ ] Device identifiers and serial numbers
- [ ] Web addresses – universal resource locators (“URLs”)
- [ ] Internet protocol (“IP”) address numbers
- [ ] Biometric identifiers, including fingerprints and voice prints
- [ ] Full face photographic images and any comparable images
- [ ] Other unique identifying number, characteristic, or code (except a re-identification code)

If Other, explain:

Please clearly describe all PHI elements that will be shared outside Geisinger and to whom (such as, FDA, sponsor, other sites, collaborators, etc.)?

No PHI will be shared outside of Geisinger.

PHI may only be used or disclosed for research purposes under one or more of the following circumstances:

i.   Written HIPAA Authorization
ii. Data Use Agreement (DUA) for the use of a limited data set
iii. Agreement for Disclosure of PHI to another institution
iv. Clinical trial or sponsored research agreement or sub-award

Waiver/Alteration of Authorization
i. Data Use Agreement (DUA) for the use of a limited data set
ii. Agreement for Disclosure of PHI to another institution

5.17 Are you requesting a Waiver of Authorization for the entire study?

○ Yes ◉ No

6.0 Written Consent

6.1 Describe the setting and conditions under which consent/assent will be obtained, including parental permission or surrogate permission and the steps taken to ensure subjects' independent decision-making.

The setting and conditions under which consent will be obtained will be in the Center for Nutrition and Weight Management. The patient will be consented in the privacy of the exam room. Patients will be able to ask questions and take consent home to review if necessary.

6.2 List all personnel who will be involved in the consent process, which includes the consent interview, etc.

Research assistants will be consenting patients.

6.3 Describe the waiting period between informing the prospective participant and obtaining consent?

If none, please provide justification.

The waiting period will be variable depending on when the patient is informed about the study.
Some patients will be informed about the study prior to surgery at a class/session or at an individual pre-op appointment therefore, the consent might not be signed for a number of weeks or months because they can not sign consent until they have had bariatric surgery.
Patients who have already had bariatric surgery will be informed about the study via a letter and then a research assistant will call the patient a week later and provide more information and set up a time for the patient to come in and sign consent if they are interested in participating. Therefore, the consent again might not be signed for several weeks

6.4 Specify the documents that will be used during the consent/assent process. Copies of all documents should be appended to the protocol, in the same format that they will be given to subjects.

A recruitment letter will be mailed to possible participants, and a telephone script will be used by research assistants to describe the research study. Research assistants will go through the informed consent during the first study visit.

6.5 Explain provisions in place to ensure comprehension for research involving non-English speaking subjects. Translated copies of all consent materials must be submitted for approval prior to use.

We will not consent non-English speaking subjects.

6.6 Indicate how the personnel obtaining consent will assess the potential subject's ability and capacity to consent to the research being proposed.

The research staff will assess the potential subject's ability and capacity to consent for the proposed research in a face to face manner which should allow for any concerns about capacity or ability to be addressed immediately. All subjects will be allowed appropriate time to independently read, review and ask questions regarding the consent form and research study in a private setting, thus lessening the likelihood of coercion and pressure to enroll.

7.0 Request for Partial Waiver of Authorization for Recruitment

7.1 *Complete this appendix to request a partial waiver of authorization for recruitment purposes.*

In the main application you indicated the PHI that will be collected as part of this research protocol. Please answer the following questions:

7.2 Explain how the use and disclosure of the information presents no more than minimal risk to the privacy of the individual?

A Request for Partial Waiver of Authorization for Recruitment is requested to identify a specific cohort within the

Geisinger system that fit the eligibility criteria for recruitment purposes. Only the research team will have access to

this information (MRN, Name, Address, Phone Number, Return Appointment Dates).

7.3 Describe the plan to protect the identifiers from improper use and disclosure (i.e., where will the identifiers will be stored and who will have access.)

Information will be stored on a password protected computer on a secure network and only the research team will

have access. All consent/authorization forms will be maintained for a minimum of 6 years following the completion

of the study.

7.4 Describe the plan to destroy the identifiers at the earliest opportunity consistent with the conduct of the research. If there is a health or research justification for retaining identifiers or if such retention is required by law, please provide this information as well.

The plan to destroy the indentifiers collected for this part of the process will be destroyed once enrollment is closed.

7.5 Explain why the research could not be practicably conducted without the alteration or waiver.

Completing enrollment in the timeframe outlined cannot be practicably achieved without pre-screening. Prescreening

involves CDIS data pull and manual chart review of patients seen within the medical center to

determine eligibility and send recruitment letters. As previously mentioned we will receive a list of names,

addresses, medical record numbers, and phone numbers, and return appointment dates for all participants that meet inclusion/exclusion criteria.

7.6 Explain why the research could not be conducted without access to and use of the PHI.

Recruitment letters will be sent to all eligible participants. This process cannot be completed without access to names, addresses and phone numbers. Chart reviews of participant eligibility cannot be made without access to medical record numbers. Patient names, phone numbers and medical record numbers will be reviewed by Geisinger study personnel for recruitment purposes and minimal chart reviews to determine eligibility. This is the minimum amount of PHI necessary to complete a letter and/or phone call to review eligibilty for this study. This recruitment method cannot be conducted without access to this information
